# Idiopathic Thoracic Spontaneous Spinal Epidural Hematoma

**DOI:** 10.1155/2016/5430708

**Published:** 2016-03-21

**Authors:** Abdurrahman Aycan, Seymen Ozdemir, Harun Arslan, Edip Gonullu, Cemal Bozkına

**Affiliations:** ^1^Neurosurgery Department, Yuzuncu Yil University Faculty of Medicine, Van, Turkey; ^2^Department of Neurosurgery, Van District Training and Research Hospital, 65100 Van, Turkey; ^3^Department of Radiology, Van District Training and Research Hospital, 65100 Van, Turkey; ^4^Department of Anesthesiology and Reanimation, Van District Training and Research Hospital, 65100 Van, Turkey

## Abstract

A 33-year-old male patient experienced temporary sensory loss and weakness in the right lower extremity one month prior to admission. The patient was admitted to a private clinic with a three-day history of acute onset of sensory loss and weakness in both lower extremities and was treated and followed up with a prediagnosis of transverse myelitis and the Guillain-Barre syndrome (GBS). The patient was subsequently transferred to our clinic and the neurologic examination revealed paraplegia in both lower extremities, positive bilateral Babinski signs, and hypesthesia below the T10 dermatome with saddle anesthesia. The patient had urinary incontinence and thoracic magnetic resonance imaging (MRI) showed an image of a mass compressing the medulla.

## 1. Introduction

Acute spontaneous spinal epidural hematoma (ASSEH) is a rare condition that requires emergency intervention in the presence of neurological deficit [1]. ASSEH was first described by Blauby in 1808 [2]. The exact etiology of ASSEH remains unknown in 40–50% of the patients although coagulopathy, epidural catheter and surgical trauma, use of anticoagulants, and the conditions causing increased intrathoracic-intra-abdominal pressure such as cough and Valsalva maneuver have been reported as the predisposing factors for ASSEH [3, 4]. The incidence of ASSEH is considered to be 0.1 per 100,000/year [1]. ASSEH is mostly presented by an acute onset of severe back pain. This pain may progress toward motor and sensory loss, depending on the level of the lesion.

The ASSEH patients with progressive neurological deficit require urgent decompression of the spinal cord and the evacuation of the hematoma [5–7]. On the other hand, there are several studies suggesting that the patients with no neurological deficit or the patients showing spontaneous recovery can be conservatively followed up [8].

In this report, we present a case who was admitted with progressive paraplegia three days after the onset of the complaints and whose diagnosis was delayed during the investigation of the neurological signs.

## 2. Case Report

**Figure 1:**
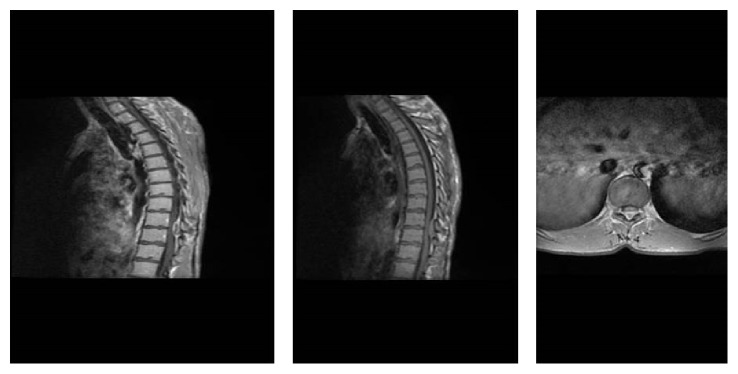
Preoperative thoracic sagittal T1, T2 MRI and axial T2 MRI.

**Figure 2:**
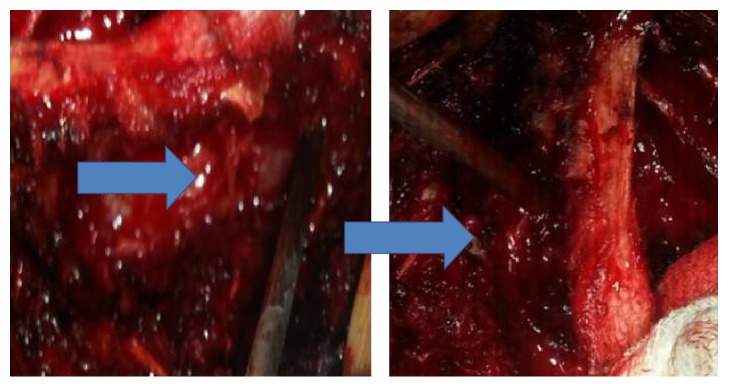
Thoracic spinal epidural hematoma images during surgery.

**Figure 3:**
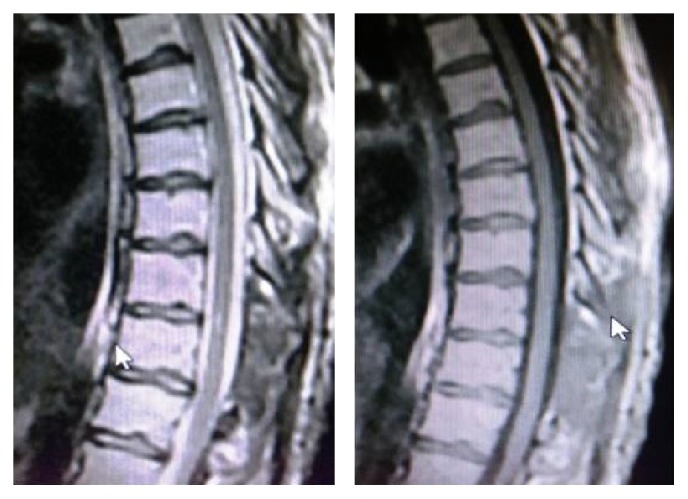
Postoperative thoracic sagittal T1-T2 MRI.

**Figure 4:**
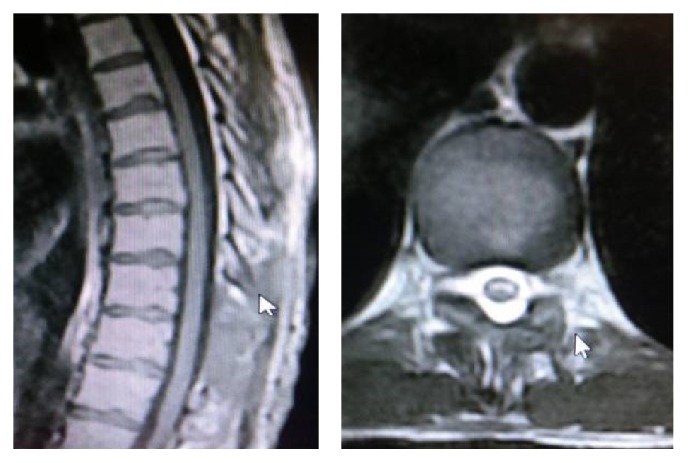
Postoperative thoracic sagittal T1-axial T2 MRI fields marked hemilaminectomy.

A 33-year-old male patient presented to a private clinic with a three-day history of acute onset of sensory loss and weakness in both lower extremities. The patient presented to our emergency service when the neurological signs were being investigated by a neurologist in the previous clinic. On admission, the patient had no history of trauma, bleeding disorder, and hypertension. The neurological examination revealed paraplegia in both lower extremities, positive bilateral Babinski signs, and hypesthesia below the T10 dermatome with saddle anesthesia. The anal sphincter tone and bilateral tendon reflex were reduced. The blood levels were in normal range and the blood coagulation tests revealed normal international normalized ratio (INR) and activated partial thromboplastin time (APTT). Cervical, thoracic, and lumbar MRI scans revealed that a hyperintense signal change on T1-weighted and an isointense signal change on T2-weighted images were compressing the spinal cord (Figure 1). The patient was operated on under emergency conditions. A right hemilaminectomy was performed since the compression was greater on the right side and no radiological differential diagnosis was performed for spinal mass, spinal abscess, and epidural hematoma. Total laminectomy was abandoned due to the intraoperative detection of the hematoma (Figure 2). Hemilaminectomy was extended and the hematoma was evacuated. The dura mater was removed through irrigation with serum physiologic. On postoperative day 1, the patient completely regained normal muscle and sensory function in the right lower extremity. On postoperative day 3, MRI signs were improved (Figures 3 and 4). On postoperative day 6, the patient had no motor and sensory loss in both lower extremities and was discharged with a complete neurological recovery and complete radiological improvement.

## 3. Discussion

Spontaneous epidural hematomas (SEHs) can be classified into two categories: traumatic and spontaneous. Traumatic SEHs are considered to occur secondary to spinal surgeries, lumbar puncture, vertebral fractures, and any spinal intervention [9]. Acute spontaneous SEHs do not present with any underlying condition in almost half of the patients, although anticoagulant treatment, arteriovenous malformation (AVM), tumors, infections, and coagulopathy are considered to be the predisposing factors. Our case was diagnosed as ASSEH since no predisposing factor or history of trauma was present and no abnormality was seen in the bleeding patterns.

ASSEH can be seen in all age groups, with the highest incidence in males and patients aged over 40–50 years. However, ASSEH is very rarely seen in children [10]. Groen [11] found no correlation between age, gender, and postoperative outcomes.

Ventral SSEH is highly rare since the dural sac is firmly attached to the posterior longitudinal ligament. Anterior SSEH has also been reported, though very rare [12]. The dorsal aspect of this space is filled with fatty tissue; therefore, posterior SSEHs are more common than anterior SSEHs. Similar to the literature, the source of bleeding in our patient was posterior.

SSEH mostly occurs in the lumbar region in patients aged over 40 years and in the cervical and thoracic segments in patients aged below 40 years and children [10]. In contrast to the literature, our case was aged 33 years and the source of bleeding was in the thoracic region.

The source of bleeding in SSEH remains unknown [13]. Although some studies suggest that SSEH may result from the rupture of the spinal venous system caused by the pressure changes within the posterior epidural venous plexus (which has thin walls and no valves) [11], some others maintain that SSEH may arise from a minimal impairment or laceration in the epidural artery caused by the low pressure within the venous plexus compared to the intrathecal pressure [14].

SSEH is mostly presented by a sudden onset of pain in the neck and back and weakness. This pain is of a radicular character and may radiate to the extremities. Within hours or days, due to the compression of the spinal cord, these symptoms may lead to varying degrees of motor and sensory loss. Diagnosis of SSEH is difficult to establish prior to the onset of neurological deficit. Therefore, the differential diagnosis of SSEH should include pulmonary emboli, spontaneous pneumothorax, and acute myocardial infarction in the absence of neurological deficit and should include transverse myelitis, GBS, epidural subarachnoid bleeding, and acute spinal cord ischemia in the presence of neurological deficit [15]. In our case, the definitive diagnosis was not established until the third day after admission since the patient was prediagnosed as GBS and transverse myelitis.

MRI remains the method of choice in the diagnosis of SSEH. MRI provides useful outcomes in the visualization of the location and the size of the hematoma as well as the presence of the spinal cord compression and edema. SSEH yields an isointense signal change on T1-weighted images within the first 24 h after bleeding and a hyperintense signal change on T2-weighted images after 24 h [1]. In our case, the bleeding was considered to be subacute, depending on the MRI scan which was performed after a 3-day delay.

Urgent decompression of the spinal cord, regardless of the cause of bleeding, in the SSEH patients with neurological deficit is the initial step to be performed for the recovery from neurological deficit and for the classification of the hematoma [16]. On the other hand, there are several studies suggesting that the patients with no neurological deficit or the patients showing spontaneous resorption or spontaneous recovery can be conservatively followed up with the aid of imaging techniques [8, 17–19].

The preoperative neurological status and the interval between the onset of symptoms and operation are key factors for the prognosis [17–20]. Moreover, the shorter the time between the onset of symptoms and the application of decompression, the better the neurological response to be received through the treatment [21–24]. Moreover, prompt decompression of the spinal cord is of prime importance because prolonged compression of the spinal cord may lead to irreversible injury for the spinal cord. In a meta-analysis, Kreppel et al. [25] reported that complete recovery from neurological deficit may be achieved if the time between the onset of neurological deficit and operation does not exceed 12 h. Conversely, Foo and Rossier [3] suggested that the time between the onset of symptoms and operation has no importance for the treatment. On the other hand, Lawton et al. [26] maintained that it is highly difficult to define the most favorable time for the application of decompression surgery. In our case, in contrast to the literature, the time between the onset of noticeable symptoms and operation was more than 72 h. However, despite this prolongation, the patient made a complete recovery.

Conventional SSEH treatment includes total laminectomy and the evacuation of the hematoma. However, we performed hemilaminectomy, which is a minimally invasive method, for the evacuation of the hematoma and we believe that this method can be preferred in order to prevent the risk of postoperative complications such as kyphotic deformities and instability.

## 4. Conclusion

ASSEH should be kept in mind in the patients presenting with neurological deficit and a sudden onset of pain in the back. Urgent decompression surgery is the mainstay treatment in the patients with ASSEH and should be performed in order to avoid the risk of adverse outcomes. Neurogenic claudication may occur secondary to the compression of the spinal cord with no invasion into the neural tissue; therefore, surgical intervention is the ideal method for achieving complete recovery. In the cases where radiological differential diagnosis is difficult to perform, the surgery should be initiated by a minimally invasive approach and the surgeons should be prepared for the surgeries that may be needed for other diagnoses. MRI is the golden standard for the differential diagnosis of the patients suspected with SSEH.

In the cases with normal neurological findings, the surgeons may prefer to wait for the resorption to occur by performing repeated examinations and radiological follow-ups. Moreover, the absence of underlying diseases is also a possible condition, though rare.
